# Effective Feature Selection Methods to Detect IoT DDoS Attack in 5G Core Network

**DOI:** 10.3390/s22103819

**Published:** 2022-05-18

**Authors:** Ye-Eun Kim, Yea-Sul Kim, Hwankuk Kim

**Affiliations:** 1 Department of Electronics Information and System Engineering, Sangmyung University, Cheonan 31066, Korea; yeni0.0king@gmail.com (Y.-E.K.); rlavnf10106@gmail.com (Y.-S.K.); 2 Department of Information Security Engineering, Sangmyung University, Cheonan 31066, Korea

**Keywords:** 5G, sensor network, machine learning, feature selection, IoT DDoS, DDoS detection

## Abstract

The 5G networks aim to realize a massive Internet of Things (IoT) environment with low latency. IoT devices with weak security can cause Tbps-level Distributed Denial of Service (DDoS) attacks on 5G mobile networks. Therefore, interest in automatic network intrusion detection using machine learning (ML) technology in 5G networks is increasing. ML-based DDoS attack detection in a 5G environment should provide ultra-low latency. To this end, utilizing a feature-selection process that reduces computational complexity and improves performance by identifying features important for learning in large datasets is possible. Existing ML-based DDoS detection technology mostly focuses on DDoS detection learning models on the wired Internet. In addition, studies on feature engineering related to 5G traffic are relatively insufficient. Therefore, this study performed feature selection experiments to reduce the time complexity of detecting and analyzing large-capacity DDoS attacks in real time based on ML in a 5G core network environment. The results of the experiment showed that the performance was maintained and improved when the feature selection process was used. In particular, as the size of the dataset increased, the difference in time complexity increased rapidly. The experiments show that the real-time detection of large-scale DDoS attacks in 5G core networks is possible using the feature selection process. This demonstrates the importance of the feature selection process for removing noisy features before training and detection. As this study conducted a feature study to detect network traffic passing through the 5G core with low latency using ML, it is expected to contribute to improving the performance of the 5G network DDoS attack automation detection technology using AI technology.

## 1. Introduction

A 5G network is a massive IoT environment with low latency [1,2]. When an IoT device with weak security is connected to a 5G network, a Tbps-level DDoS attack targeting the 5G mobile network occurs, resulting in a network failure (delay) [3]. This can cause major security problems for 5G core network functions and devices connected to the networks. Machine learning (ML) models learn the rules for making intrusion decisions. Building an automated intrusion detection system using ML can solve the time and cost limitations. In addition, generalized performance can be guaranteed for new attack patterns. Therefore, interest in the automation of intrusion detection using ML in 5G networks is increasing. However, DDoS attack detection using ML in 5G networks is limited by the processing of large amounts of data generated by 5G devices. Using all available features as input can lead to ML models performing poorly and wasted training and detection time. Therefore, the massive amount of 5G data raises the problem of selecting key features related to real-time learning and detection to provide ultra-low latency. To detect large amounts of traffic in 5G networks in real time, determining fewer important features while ensuring the learning performance is necessary. Thus, the feature-selection process can be used. In ML classification problems, the feature selection process is an essential preprocessing step for identifying important or highly relevant features in large datasets. The feature selection process maintains and improves performance while reducing computational complexity by removing noisy features that are less relevant to learning [4,5,6,7]. This feature selection method enables ultra-low-latency 5G services through real-time DDoS attack detection using an ML algorithm. Therefore, research on features that can reduce the time complexity for the real-time detection of large-capacity DDoS attacks in a 5G mobile network environment is required.

A 5G network consists of a service-based architecture (SBA) that is subdivided and virtualized into network function (NF) units. The International Telecommunication Union Telecommunication Standardization Sector (ITU-T) and 3rd Generation Partnership Project (3GPP) are researching the internalization of security in the SBA of 5G networks, a 5G E2E security framework, including SBA security in the SG17 core network security of ITU-T [8], and 5G SBA NF security research conducted by 3GPP [9]. In addition, as artificial intelligence (AI) technology advances, it is drawing attention as a major technology in 5G network design. To implement AI-based 5G network automation in 3GPP and optimize the related NF, a network data analysis function (NWDAF) was introduced in the 5G core network to adopt the network design method for AI [10,11]. Accordingly, prior research is necessary to implement security functions using the AI-native core architectures of 5G and 6G networks [12].

Previous studies on the performance improvement of DDoS attack detection using ML and feature selection have focused on the wired network environment [13,14,15,16,17]. Therefore, to provide ultra-low latency service in a 5G mobile network environment, studying the feature engineering of DDoS attack detection related to time complexity reduction is necessary. Thus, we constructed a 5G tested environment for experiments, collected GPRS Tunneling Protocol (GTP) packets by establishing hypotheses, explained DDoS detection results using feature selection, and presented future studies.

In this study, to detect large-scale DDoS attacks using ML in real time in a 5G mobile network environment, feature selection experiments that can reduce time complexity were performed, and the results were analyzed. A virtual 5G environment (UERANSIM + Open5GS) was constructed to collect GTP-U packets, i.e., 5G datasets from the user plane by reapplying the Kitsune dataset [18,19] on a 5G network. The 5G dataset collected was multiclass classified using the ML algorithms decision tree (DT), random forest (RF), k-nearest neighbors (KNN), and Stacking Ensemble. To compare their performance, various feature selection methods were used, namely, activating all features and activating selected features. In addition, the difference in the required time was compared while increasing the size of the data.

The main contributions of this study can be summarized as follows. First, we noted the importance of removing extraneous features to detect IoT DDoSs with low latency in the 5G core network path. The experiments show that the feature selection process can lead to faster and better classification models by selecting features that have a large influence on learning and detection. Second, we constructed an experimental 5G mobile environment and collected datasets using multiple user equipment (UE). Third, we evaluated the results of feature selection by classification algorithms and presented all performance indicators related to our experiments. Finally, we showed the usefulness of the feature selection process in terms of low-latency detection of IoT DDoS attacks on massive 5G datasets. Subsequently, we confirmed the significance of the feature selection study for the real-time detection of DDoS attacks in an ultra-low latency 5G core environment.

The remainder of this paper is organized as follows. Section 2 presents previous studies related studies on 5G network attack detection and feature selection. Section 3 describes the concepts of 5G system architecture and feature selection. Section 4 defines the problem and outlines the IoT DDoS detection methodology of 5G environments employed in this study. Section 5 presents the results of the feature selection method. Section 6 compares the results of using all features extracted with the results obtained using the feature selection process. By increasing the size of the dataset, it shows the effect of the feature selection process on the detection of a large amount of IoT DDoS. Finally, Section 7 provides concluding remarks regarding this study and directions for future research.

## 2. Related Work

### 2.1. Wired Network Environment

Studies related to malicious traffic detection using ML and feature selection in wired networks are as follows. Babaagba et al. [13] conducted a best detection model study using feature selection for anomaly detection with supervised ML. A total of 149 samples were collected and analyzed, including 81 clean and 68 malware samples. Six types of supervised ML and one type of unsupervised ML were used in this study. The accuracy of each algorithm was as follows: random forest (RF), 73.15%; decision table, 76.51%; Bayesian network, 68.45%; multilayer perceptron (MLP), 77.18%; lazyIBK, 74.49%; logitboost, 76.51%; and EM, 54.36%. Among them, the MLP algorithm was proposed as an effective detection approach.

To improve the accuracy of an ML classifier and detect malicious traffic, Shafiq et al. [14] presented a feature selection model that uses WMI_AUC and RFS. Two datasets were used for an experimental evaluation, namely, HIT Trace 1 and NIMS, developed in separate networks. Furthermore, 11 types of ML classifiers were used, i.e., Bays Net, naïve Bayes, SMO, AdaBoost, Bagging, OneR, PART, Hoeffding, C4.6, RF, and random tree. The experimental results showed that decision tree ML classifiers, namely, RF and C4.5, performed efficiently when testing with features selected using WMI_AUC and RFS.

Idhammad et al. [15] presented an online sequential semi-supervised ML approach based on a network entropy estimation, co-clustering, information gain ratio, and extra trees algorithms for DDoS detection. Three datasets (NSL-KDD, UNBISCX12, and UNSW-NB15) were used to evaluate this approach, and the results showed the accuracy and false-positive rates. The accuracy for each dataset was 98.32% for NSL-KDD, 99.88% for UNBISCX12, and 93.71% for UNSW-NB15. The false-positive rates were 0.33%, 0.35%, and 0.46%, respectively.

Alzahrani et al. [16] and Seo et al. [17] proposed ML-based IoT network anomaly detection models that can be applied to an IoT gateway or device in a wired network. Specifically, Alzahrani et al. [16] proposed a real-time lightweight model to classify traffic using random undersampling and word embedding algorithms, whereas Seo et al. [17] proposed a framework with an ML-based botnet anomaly detection IoT device and sequential extraction architecture. In the experiment, most of the relevant features were selected and configured for each attack class.

As mentioned above, several studies have used ML and feature selection in wired environments, and each has shown significant results. However, because GTP traffic is not analyzed, their application is limited for detection in 5G core networks.

### 2.2. 5G Network Environment

#### 2.2.1. Using GTP Tunneling Packet

Kim et al. [20] conducted a study on ML-based IoT botnet traffic detection in a 5GC network path using binary classification for detecting benign and malicious traffic and multiclass classification for benign and malicious traffic types. All 5G Core GTP packets were used, and free5GC, an open-source project for 5th generation (5G) mobile core networks, was used to configure the GTP tunneling testbed. MedBIoT was used as the dataset, labeled “benign” or “malicious”, and the GTP packet was generated by dumping the packet into the free5GC testbed. Four algorithms were used for anomaly detection (KNN, SVM, RF, and stacking). When evaluating ML performance, IP+GTP packets were used simultaneously for IoT botnet detection in a 5GC environment to obtain performance comparable to that of a wired network. The results showed that stacking achieved 99.924% in binary classification and 97.5% in multiclass classification.

Ref. [20] performed detection using GTP-U packets collected from the 5G core network path; however, high performance was obtained using both IP and GTP packets. In addition, all features were used without selecting significant features during feature selection. Therefore, ref. [20] demonstrated a high performance with high time complexity.

#### 2.2.2. 5G Network Environment with ML

This section outlines research on anomaly detection using ML in 5G mobile networks. Alamri et al. [21] analyzed appropriate ML models to protect 5G SDN controllers, which are targeted in DDoS attacks. The datasets used were CICDDoS201 and NSL-KDD, and the algorithms were XGBoost, RF, and logistic regression. The experimental results indicate that XGBoost is the most effective ML model.

Based on a software-defined 5G architecture, Li et al. [22] proposed an IDS intelligence with improved software-defined technology and AI. The detection performance improved through balanced dataset training. The selected combination of ML algorithms (K-means++ and Adaboost) was also more effective than the existing solution. The datasets used were NSL-KDD and KDD Cup 1999.

Polat et al. [23] presented an ML-based DDoS attack detection model for 5G SDN networks. The dataset was used to collect specific features from DDoS attacks and normal traffic in an SDN. Four ML algorithms, i.e., SVM, NB, ANN, and KNN, were used to compare scenarios with and without feature selection. The experimental results showed that KNN with wrapper FS achieved the highest performance with an accuracy of 98.3%.

Refs. [21,22,23] performed DDoS detection using ML in a 5G SDN environment. Refs. [22,23] proposed an effective detection model using feature selection. However, this study constructed a virtual 5G core network environment, not an SDN environment. In addition, detection was performed by directly transmitting packets from the UE to the 5G core NF.

#### 2.2.3. 5G Network Framework with ML

This section describes studies that have proposed new 5G frameworks with improved ML-based anomaly detection. Monge et al. [24] proposed FlowSentinel, a new 5G anomaly detection framework based on SELENET. This study uses ETSI-NFV, a 5G network environment. Using the ML model in a 5G environment, the model analyzes the outbound traffic flow to identify the characteristics of malicious activities and devices attempting flooding-based DDoS attacks. The ML algorithms used to analyze the traffic flow were RF and classification and regression trees (CART). The dataset used designated labels for training and testing purposes, and used the traffic collected from 61 devices with different characteristics. Considering the traffic dataset observed in an actual device, preliminary observations indicate that normal activities were effectively distinguished from DDoS activities of varying intensities.

Maimo et al. [25] presented a 5G-oriented architecture to identify cyberthreats using deep learning (DL). This study used ETSI-NFV, which is a 5G network environment. LSTM was used as the DL algorithm, whereas the CTU dataset (a botnet dataset) was applied to analyze 5G anomaly detection. The results were acceptable for traffic evaluation and classification under 5G scenarios.

Refs. [24,25] is a 5G network model based on ETSI and SELENET. In this study, the differences in feature selection performance were examined according to various ML algorithms for anomaly detection in a 3GPP-based 5G mobile network environment.

## 3. Background

### 3.1. 5G System Architecture

**Figure 1:**
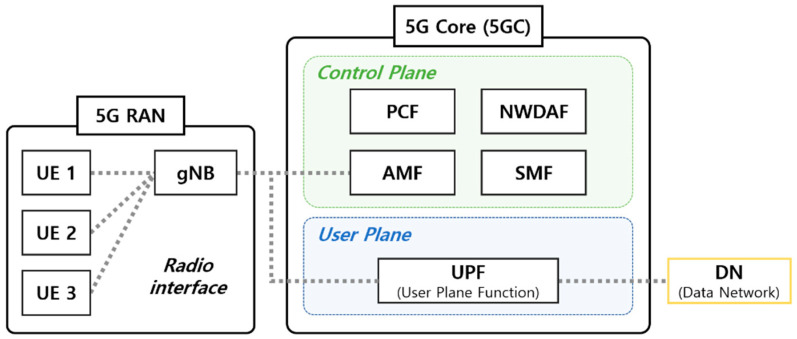
The 5G system architecture.

The 5G system architecture (5GS) includes a 5G access network (AN), 5G core network (5GC), and UE [26] (Figure 1) and is defined as service-based. The 5G (R)AN provides a wireless interface for the UE. The 5G base station (gNB) of the (R)AN provides the GTP to the UE. GTP is a tunneling protocol that is defined to carry data within mobile networks. The network packet generated from the UE is transmitted to the 5GC via GPRS tunneling through the (R)AN. GTP consists of a control plane (GTP-C), user plane (GTP-U), and charging (GTP’ derived from GTP-C) traffic [27].

Several NFs in the 5GC provide services through the serviced-based interface (SBI) of the SBA. The 5GC network is responsible for functions such as session management, mobility, authentication, and security [28]. The control plane is responsible for control, and the user plane is responsible for data packet transmission [29]. In the control plane, mobility is mostly managed through AMF and SMF in 5GC. The AMF manages the access and mobility of UEs through location service messages. The SMF allocates IP addresses to the UEs and manages user plane services [30]. PCF defines policies of all types in the network and provides them to NFs (e.g., AMF, SMF, et al.) in other control planes [31,32]. Each NF of the 5GC constituting the SBA controls data transmission [33]. The 5GS was enhanced to support network data analysis services through the NWDAF [34]. NWDAF collects and analyzes information on various network domains. It is a core functional entity that provides analysis-based statistical and predictive insights into 5GC. To perform tasks such as mobility prediction and optimization, DDoS attack detection, and predictive QoS and data correlation, ML algorithms can utilize multiple network information collected through the NWDAF [35]. The user plane function (UPF) of the 5GC user plane is connected to the data network (DN) and is responsible for packet routing and forwarding.

### 3.2. GPRS Tunneling Protocol in 5G

This study collected GTP tunneling packets to be transmitted to a 5G core and detected DDoS attacks through them. Therefore, the GTP uplink process for a network packet in 5G is described.

**Figure 2:**
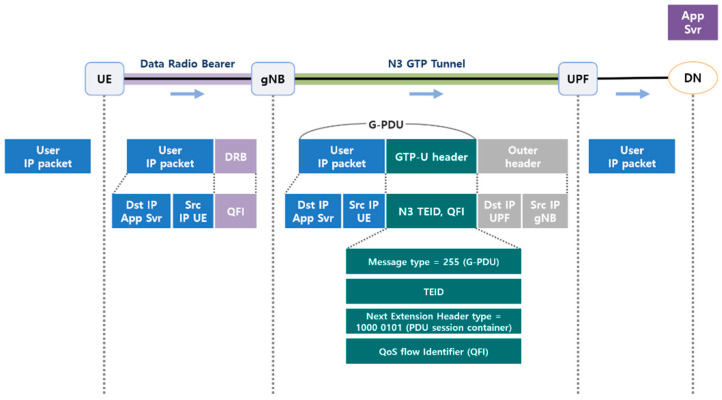
The 5G network flow—Uplink (Adapted with permission from Ref. [36]. 2019, Netmanias).

Figure 2 shows the uplink flow through the user plane of the 5G network traffic. The IP packet generated by the UE is forwarded to the gNB for transmission to the DN. When the gNB receives an IP packet, it encapsulates the IP packet in the GTP-U header. Subsequently, the packet is encapsulated inside the IP and UDP headers and is then transmitted to the UPF. The encapsulated IP packet and GTP-U header make up the G-protocol data unit (G-PDU). The GTP-U header consists of the message type, next extension header type, tunnel end-point identifier (TEID), and QoS flow identifier (QFI). The message type is fixed to decimal number 255 (0xff), indicating the G-PDU, and the next extension header type is fixed to binary number ‘1000 0101’ (0x85), indicating the PDU session container. The transmitted packet in the QoS flow is identified by the QoS flow identifier. TEID is an identifier uniquely assigned to each GTP user connection on each node [12].

### 3.3. Feature Selection

Feature selection is an important technique for the efficient learning and operation of ML models. It is used to reduce overfitting and learning time as well as to improve accuracy. Feature selection is classified as unsupervised or supervised according to the value of y (label). Supervised feature selection selects features based on the label and is largely divided into three types of methods, i.e., filter, wrapper, and embedded methods [4].

**Filter Method.** A filter method uses a statistical measurement technique to determine the influence and correlation of a feature, as opposed to that of the best feature subset. Hence, each feature is considered an independent variable. This method had the fastest speed in terms of time complexity. Some examples include feature importance and statistical methods.

**Wrapper Method.** A wrapper method determines a subset with the optimal usefulness by comparing subset combinations of all features in a specific ML algorithm. Therefore, the more features that exist, the higher the number of computations involved; thus, the method has the slowest speed in terms of time complexity. However, this improves the performance of the model because it determines the best feature subset. When the purpose of the ML algorithm is classification, the subsets are evaluated and compared based on their accuracy, precision, recall, and F1-score. Examples include recursive feature elimination (RFE), recursive feature elimination (RFECV), and sequential feature selection (SFS).

**Embedded Method.** An embedded method is used to learn features that contribute to the accuracy of the model. In terms of time complexity, this approach is between the wrapper and filter methods. Examples of embedded methods include LASSO, elastic net, and ridge regression.

In this study, feature selection was applied to find a small number of feature combinations (5 or 10) that maintained or increased the 5G malicious packet detection rate compared with a scenario in which all 55 features were used. The feature selection methods used in the experiment were the filter (feature importance) and wrapper (RFE, RFECV, SFS) methods. Feature importance is a method that uses the importance value provided by a tree-based model. RFE repeats the training using all features by removing features with the lowest importance until a user-specified number of features remains (the default is half of all features). RFECV derives the best feature subset using the same process as RFE. However, unlike RFE, the number of features can be unspecified, and cross-validation is possible. SFS finds the best feature subset by adding or removing features using the forward or backward method. SFS must specify the number of features for selection, and cross-validation is also possible.

## 4. Problem Define and Experimental Design

### 4.1. Problem Statement

The purpose of this paper is to demonstrate and investigate feature selection as a framework to detect DDoS in 5G network traffic. We explain it in two parts: defining the problem statement and presenting the objective function of this experiment. Finding the most influential features related to 5G network traffic can be formally given as follows:

Let X be a dataset containing n pairs (*x*_i_, *y*_i_) of 5G network traffics, where *x*_i_ ∈ X is a data sample described by m input features, and target variable *y*_i_ corresponds to its multi-class.

X = {(*x*_1_, *y*_1_), (*x*_2_, *y*_2_), …, (*x*_n_, *y*_n_)}, where n is the number of samples *y*_i_ ∈ {*y*_1_, *y*_2_, …, *y*_k_}, where k is the total number of classes (1)

Assume that feature vector *F* denotes a full feature set, *fi* is a feature, and selected feature subset vector *S* denotes the set of subset variables, respectively. The first objective function *g*(*x*), identifies irrelevant, redundant features in 5G networks and finds the best representative subset of features to improve machine learning accuracy. Given a feature set *F =* {*f_i_* | *i* = 1…*m*} = {*f*_1_, *f*_2_, …, *f_m_*}, find a subset *S_n_* = {*f*_1_, *f*_2_, …, *f_n_*}, with *n* < *m*, that optimizes an objective function *g*(*x*), where m is the number of input features and n is the number of subset features, respectively. Therefore *g*(*x*) maximizes the learner’s ability to classify the target class.

(2) $$S_{n}=\arg\underset{f_{i}\;\in \;F}{\max}\left(g\left(f_{i};Y;M\right)\right),\;\mathrm{where}\;M\;\mathrm{is}\;\mathrm{leanerd}\;\mathrm{model}\;$$

The second objective function, Complexity (*F*, *t*, *M*), minimizes the runtime of the training phase to reduce the cost of detecting DDoS attacks. For each feature selection method, the runtime (*t*) of the training phase can be measured to analyze the massive volume of DDoS traffic and compare detection costs.

*C*(*F*, *t*, *M*) = arg min[*C*(*F*, *t*, *M*) − *C*(*S_n_*, *t*, *M*)] (3)

where *F* is a full feature set, *S* is a selected feature set, *M* is the learner, and *t* is the training time (second) of the training data (D).

### 4.2. Experimental Design

**Figure 3:**
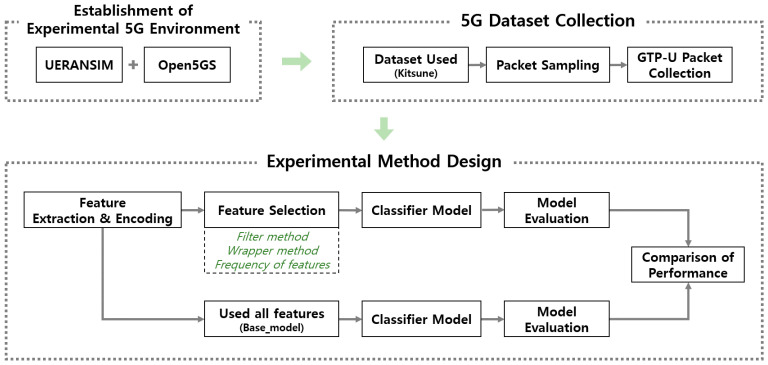
Experimental design for DDoS attack detection in a 5G mobile network.

The experiment was conducted to establish a 5G environment, collect GTP packets, and detect DDoS attacks based on ML using GTP packets. This is shown in Figure 3, which is a structural diagram of the experiment. The subsections below describe each step of the diagram in detail.

#### 4.2.1. Establishment of Experimental 5G Environment

**Figure 4:**
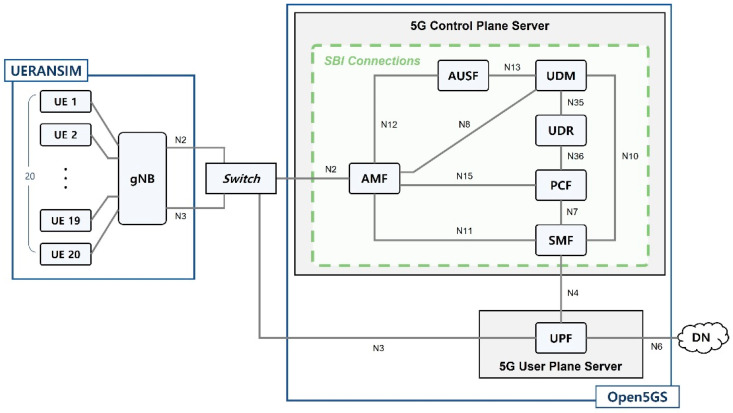
Experimental 5G environment of a 5G mobile network (UERANSIM + Open5GS).

The environment for collecting 5G data was constructed using UERANSIM and Open5GS (Figure 4); the latter was used to implement a virtual 5G core network. Open5GS can build a private network only if the gNB, eNB, and USIM are available. Thus, the UE and gNB were constructed using UERANSIM, which supports 5G RAN control and user planes. The GTP packets to be used in the experiment were collected by dumping the switch equipment between the gNB and UPF.

#### 4.2.2. 5G Dataset Collection

The 5G datasets were collected by dumping the switch equipment between the gNB and the UPF in the experimental environment described in Section 4.2.1. The assumptions and detailed processes for collecting these datasets are described in the following subsections.

(1) Dataset Used (Kitsune)

This study utilized the Kitsune Network Attack Dataset [18,19] developed at Ben-Gurion University in Israel in 2018 (updated up to 2020). The Kitsune dataset provides a total of 21,017,596 network packets for 9 individual attacks among 4 types of real network intrusion attacks (Table 1). The Kitsune dataset consists of network packets of similar IPs (unlike other datasets that use different IPs) for each attack on normal and malicious traffic. In [19], three file types for each attack are provided: extracted features (CSV files) extracted using an afterimage feature extractor [18], labeled packets (CSV files), and the original network packets (PCAP file).

This study used TEID features influenced by source and destination IPs. Hence, the Kitsune dataset was used, which consists of similar IPs for each attack. Furthermore, a PCAP file and a CSV file with labeled packets were used to relay the packets in the 5GC network.

(2) Packet Sampling

Two out of the nine attacks (Mirai, Video Injection) from [19] were excluded for the purpose of this experiment. Hence, the seven attacks utilized in this study were OS Scan, Fuzzing, ARP MitM, Active Wiretap, SSDP Flood, SYN DoS, and SSL Renegotiation.

**Figure 5:**
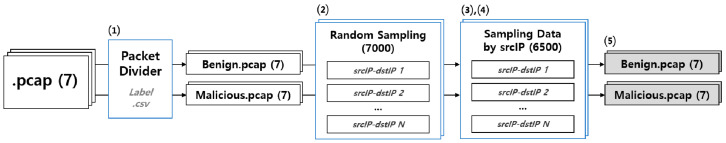
Packet sampling process.

To configure the same environment (i.e., number of network packets used) for each attack, SYN DoS with the smallest number of network packets was applied as the standard. The number of sampled network packets was set to 6500 to account for possible losses during the process of classifying benign and malicious packets in a PCAP file. Furthermore, all source and destination IP pairs were taken into consideration to reduce the dataset bias that may occur during the sampling process. In addition, to avoid biased results, the number of benign to malicious network packets per attack was matched in a 1:1 ratio. The process of sampling 6500 packets, as shown in Figure 5, is described below.

1. Based on the CSV file labeled per packet, benign and malicious packets are separated from the original PCAP file and saved as a separate PCAP file.
2. Up to 7000 packets for each source and destination IP pair in benign and malicious PCAP files saved in step 1 are randomly sampled and stored.
3. Using the source and destination IP sampling packets saved in step 2, the sampling data are saved based on the source IP.
4. A total of 6500 network packets, including all types of source and destination IP pairs, are sampled using sampling data based on the source IP (saved in step 3).
5. Consequently, a PCAP file is generated for each benign or malicious packet attack for a total of 14 PCAP files.

(3) GTP-U Packet Collection

To collect GTP packets under the most realistic 5G network conditions, the test environment described in Section 4.2.1 was constructed. Moreover, four hypotheses were made: First, a private network has a single gNB and core. Second, more than one UE may exist in a private network. Third, all UEs are connected to one gNB to communicate with a 5G core. Fourth, one UE is allocated for each source IP of the network packet.

UEs were allocated for each source IP of the Kitsune dataset packets according to these hypotheses. The sampled device level packets described in “Packet Sampling” of Section 4.2.2 were relayed from each UE to a 5G core. Subsequently, GTP-U tunneled packets were dumped and collected at the switch between the gNB and UPF (switch in Figure 4). The IPs assigned to each UE of the private network and uplink TEIDs are listed in Table 2. The UE numbers in Table 2 are expressed as “gNB number-UE number”. During the experiment, 13,000 GTP-U tunneling packets were collected for each attack, 6500 of which were benign and 6500 of which were malicious. Accordingly, 91,000 (45,500 normal and 45,500 malicious) GTP-U tunneling packets were collected.

#### 4.2.3. Experimental Method Design

(1) Feature Extraction and Encoding

The GTP-U packets described in Section 4.2.2 were collected in a PCAP file format. Therefore, the header information at the GTP-U packet level was extracted using Tshark. Headers with values were considered as features and saved in a CSV file format. Table 3 lists the features extracted using each packet protocol. Among the 55 extracted features, object-type features were encoded, and the IP address was converted into an integer after removing the decimal point. Features in the form of hexadecimal strings were also converted into decimal integers. All encoded features were input into the feature selection process to identify the smallest possible number of feature combinations while maintaining or increasing detection performance.

(2) Feature Selection

The purpose of the feature selection step is to determine the smallest number of feature combinations to achieve effective 5G malicious packet detection. This study utilized the Scikit-learn library for the filter (feature importance) and wrapper (RFE, RFECV, and SFS) methods. Each feature selection method was performed for all supported classification algorithms. RFECV and SFS, whose cross-validation can be specified, were applied using stratified K-folds as in the training and classification stages. Furthermore, for those algorithms that can specify the number of features required for selection (feature importance, SFS, and RFE), 5 or 10 features were specified for a performance comparison according to the number of features.

(3) Classifier Model

This malicious network detection experiment was conducted using four classification algorithms: DT, RF, KNN, and a stacking ensemble. The regression model of the stacking ensemble algorithm used DT, RF, KNN, and meta-regressors used logistic regression. Each ML classification algorithm was implemented using Python’s Scikit-learn library and trained using cross-validation to prevent overfitting. Stratified K-fold (n_splits = 7) was used to include all label values in the training set. Classification was conducted in a multiclass manner. Multiclass classification was applied to categorize packets into benign (0) or seven types of attacks. The labels assigned to these attacks are as follows: ARP_MitM (1), Active_Wiretap (2), Fuzzing (3), OS_Scan (4), SSDP_Flood (5), SSL_Renegotiation (6), and SYN_DoS (7).

(4) Model Evaluation

The model described in this paper was evaluated based on its ability to appropriately classify GTP-U packets. Hence, among the various available indicators, accuracy, precision, F1-score, and recall were used. In addition, the AUC and ROC curves were obtained through macro-averaging once the predicted values were one-hot encoded for each class using a one-vs-one method. The evaluation index of multiclass classification was calculated by converting the predicted result into a binary classification.

(5) Comparison of Performance

Finally, this study compared the evaluation metric results of the base model using all 55 features and the model using the features selected through each feature selection method. Furthermore, by increasing the size of the dataset, we check the effect of the feature selection process on the reduction of time complexity.

## 5. Experimental Results

Section 5 presents and compares the classification results for each feature selection method according to the experimental design described in Section 4. The results of the base model using all 55 features and the model using various feature selection methods are described in the subsections below. This study was conducted in the following experimental environment: Windows 10 Platform. Intel Core i9-10980XE (3.0 GHz/24.75 MB), DDR4 32 GB PC4-25600*4(128 GB), NVDIA GeForce RTX 3090 24 GB.

### 5.1. Base Model (Using All Features)

Table 4 shows the evaluation results of the classification using the algorithm described in Section 4.2.3, applying all 55 extracted features indicated in Table 3. As a result, KNN, an instance-based learning method, achieved the lowest accuracy of 70.038% and an F1-score of 77.96%. In contrast, the stacking ensemble, RF methods, and ensemble learning methods achieved higher scores. Stacking showed the highest score, with an accuracy of 97.264% and an F1-score of 96.991%. The base model tested in this section was then compared with the experiment using feature selection, as described in the following subsections.

### 5.2. Feature Selection Using Filter Method

#### Feature Importance

The feature importance supported by the tree-based model was used with DT and RF among the four classification algorithms in this study. Table 5 shows the multiclass classification evaluation results using the top 5 or 10 features with large importance values derived from the feature importance. It also shows the scores for each algorithm when using all 55 features. According to the experiment, both the DT and RF classification algorithms performed better with 10 features than with 5. When using the 10 features selected by the feature importance method, all scores, including the accuracy and F1-score, increased compared with the base model for both the DT and RF algorithms.

### 5.3. Feature Selection Using Wrapper Method

#### 5.3.1. Recursive Feature Elimination

RFE is a feature selection method supported by linear or tree-based models that assigns weights, such as feature importance and coefficients, to the features. Hence, among the four classification algorithms used in this study, DT and RF were applied. Table 6 shows the multiclass classification evaluation results of each algorithm using a subset of 5 or 10 features selected through RFE, as well as using all features. The results showed that high scores were recorded for the DT algorithm using five features and the RF algorithm using 10 features. When using the five features selected by the RFE method, the accuracy and F1-score of the DT algorithm increased relative to those of the base model. In contrast, the accuracy and F1-score of the RF algorithm were reduced by 1.07% and 0.82%, respectively, when using the 10 features selected by the RFE method. This is a relatively minor decrease compared with the ratio of the decrease in the number of features (approximately 82.82%), indicating the usefulness of the features selected by the RFE method.

#### 5.3.2. Recursive Feature Elimination with Cross-Validation

Similar to RFE, RFECV is a feature selection method supported by linear or tree-based models that assign weights to the features. Hence, DT and RF were used among the four classification algorithms applied in this study. By maintaining a stratified K-fold, as in the training stage, the feature subset with the best performance was extracted based on the cross-validation score. Table 7 shows the number of features of the feature subset derived through RFECV as well as the classification evaluation results using the subset. It shows the score for each algorithm using the base model described in Section 5.1. A total of 41 features were selected for the DT algorithm as the subset with the greatest influence on classification, whereas 39 features were selected for the RF algorithm. When the 41 features selected by the RFECV method were used by the DT algorithm, all scores, including the accuracy and F1-score, were higher than those of the base model. When 39 features selected by the RFECV method were used, the RF algorithm slightly increased in accuracy, but decreased in the F1-score. That is, compared with the base model, the F1-score decreased by 0.09%, and the number of features decreased by 29.09%. This result confirms the usefulness of the features selected using the RFECV method.

#### 5.3.3. Sequential Feature Selection

SFS, which compares the combination of all feature subsets, was used in the DT, RF, and KNN algorithms. Among the classification algorithms in this study, it uses a single model. In this study, SFS feature selection was applied using both forward and backward selections. Using the same stratified K-fold method as that in the training stage, the best-performing feature subset was extracted based on the cross-validation score. Table 8 shows the classification evaluation results when using a subset of 5 or 10 features selected through the SFS. The table also shows the scores for each algorithm when all 55 features are used. When applying forward selection, the results of the experiment indicated that the DT and RF algorithms performed better with five features, whereas the KNN algorithm performed better with 10 features. In backward selection, the DT algorithm performed better with 10 features, and the RF and KNN algorithms performed better with 5 features. When the features for each selection method of the classification algorithm were used, all three classification algorithms (DT, RF, and KNN) increased in accuracy and F1-score compared with the base model.

### 5.4. Stacking Ensemble with Feature Selection

The stacking algorithm described in this study uses DT, RF, and KNN as regression models. Hence, the top 5 or 10 features were input into the stacking algorithm by counting the frequency of the features selected for each classification algorithm through feature selection methods such as feature importance, RFE, RFECV, and SFS. A list of the 5 or 10 features used in the stacking algorithm is presented in Table 9. This shows that the features present in the five-feature subset were also present in the 10-feature subset. Table 10 shows the multiclass classification evaluation results using the five or ten features shown in Table 9. Based on three evaluation metrics (i.e., accuracy, precision, and F1-score), the performance was better when using 10 features than when using 5 features. When using 10 features, the accuracy decreased by 0.08%, whereas the number of features decreased by 82.82%. This indicates that the feature selection method based on the frequency of features selected for each classification algorithm used as the regression model is meaningful in the stacking algorithm.

## 6. Analysis of Experimental Results

The results obtained in Section 5 demonstrate that the features selected for each feature selection method and classification algorithm are different, and the evaluation index performance differs according to the number of features used. In this section, we compare the base model of Section 5.1 and the best performance with the classification algorithm of each feature selection method. We also compared the results with related works that are similar to this experiment. In addition, by increasing the amount of data, the time complexity change according to the feature selection application is checked and analyzed.

### 6.1. Comparison of Performance

#### 6.1.1. Comparison with Base Model

**Figure 6:**
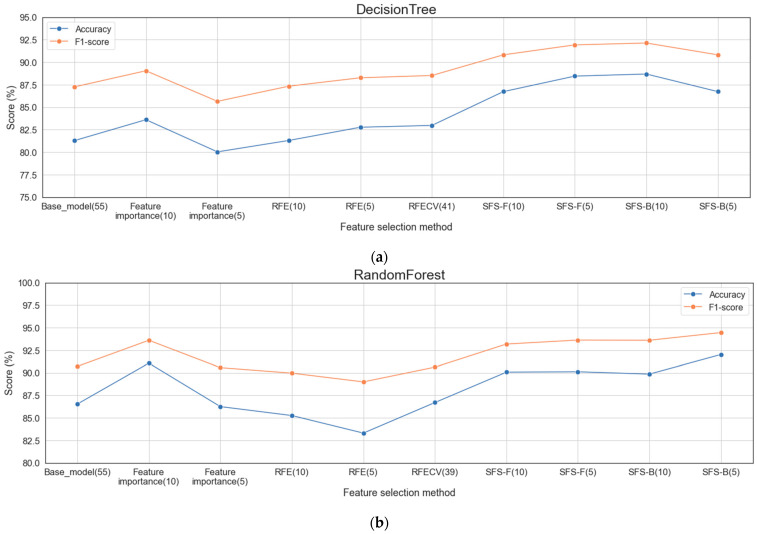

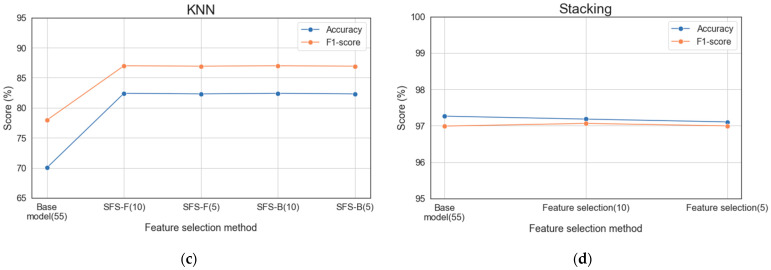
Accuracy and F1-scores of each classification algorithm for the base model and the model applying each feature selection method: (**a**) DT; (**b**) RF; (**c**) KNN; (**d**) Stacking.

**Figure 7:**
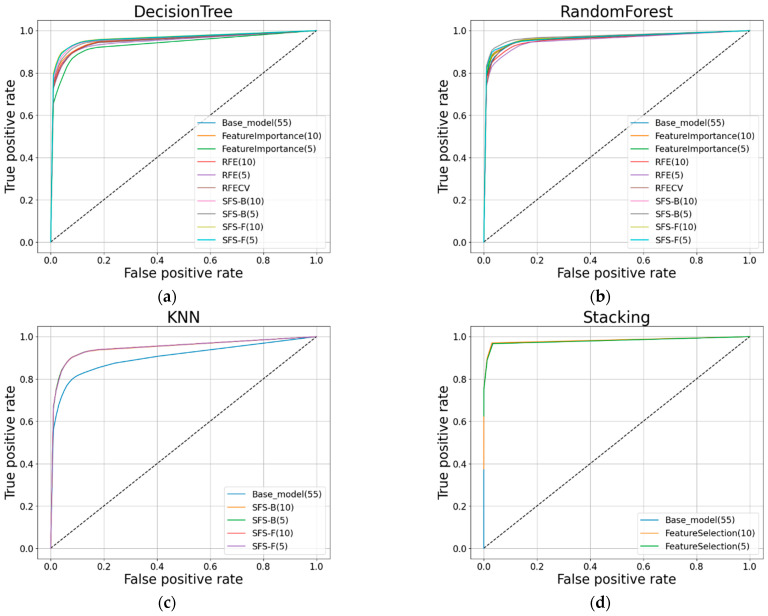
ROC curves of each classification for the base model and the model applying each feature selection method: (**a**) DT; (**b**) RF; (**c**) KNN; (**d**) Stacking.

Figure 6 shows the accuracy and F1-score of each classification algorithm for the base model using all features and the model applying each feature-selection method. Figure 7 shows the ROC curves for the base model and each feature selection method for each classification algorithm. Table 11 shows the score when using all features for each classification algorithm and the score when using the feature subset, showing good performance for each feature selection method. The multiclass classification result using all features was approximately 70.038%, which is significant, but the performance result increased with the feature selection method. In particular, the backward SFS method performed best for the tree-based classification algorithms, DT and RF. With KNN, the forward SFS method performed best according to the evaluation metrics, excluding the accuracy. The DT, RF, and KNN algorithms, which were learned by selecting the features for effective training for each classification algorithm, increased the accuracy by 9.1%, 6.34%, and 17.67%, respectively, compared to the base model. However, the accuracy of the stacking algorithm with the feature selection method decreased by 0.083%, whereas the F1-score increased by 0.076% compared with the scores when using all features.

As a result, when feature selection was used, the performance was maintained and improved. In addition, the features that had a significant influence on the classification results varied for each classification algorithm. The classification using feature selection achieving the best performance for each classification algorithm shows a lower score than that of the stacking algorithm using the feature selection method based on frequency. This indicates that the classification performance is highly dependent not only on the combination of features, but also on the classification algorithm applied.

#### 6.1.2. Comparison with Related Work

Table 12 presents the results of related studies similar to ours. Our results (Table 11) generally show good performance. In particular, the stacking algorithm, which showed the highest score among our results, showed the best performance, except for KNN in [23]. However, it should be noted that similar studies have used different datasets and models. Therefore, justifying the comparison results is difficult.

### 6.2. Comparison of Time Complexity

**Figure 8:**
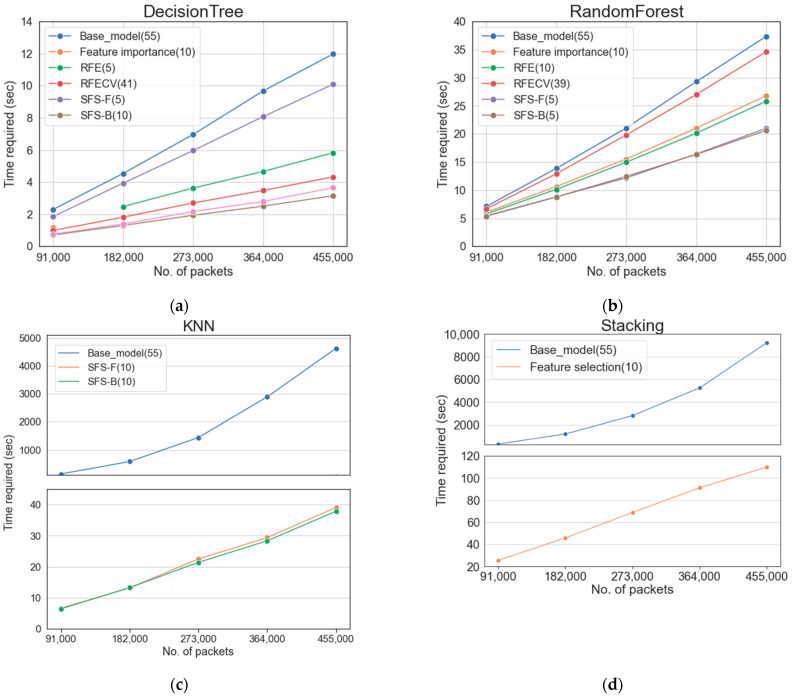
Time complexity by dataset size of the base model and the best model applying each feature selection method: (**a**) DT; (**b**) RF; (**c**) KNN; (**d**) Stacking.

Figure 8 and Table 13 show the time complexity change according to the dataset size change. When all features were used, the required time increased remarkably as the dataset increased. In particular, KNN, which compares all data, and stacking, which is an ensemble technique of several models, showed the time required in units of the hour as the data increased. However, both algorithms maintained the required time in units of seconds when using features selected for feature selection. In particular, the stacking algorithm, which showed the best performance, showed a greater rate of change (between the base model and the model using feature selection) as the dataset increased. When 364,000 packets were used for the stacking algorithm, the time required for the base model was increased by 57.6 times compared to the time required for the model using feature selection, and in the case of using 455,000 packets, it increased by 84.07 times. As a result, as the dataset increases exponentially, the difference in time complexity between the base model and the model to which feature selection is applied will gradually increase.

The time complexity change experiment, according to the dataset size change, shows the effect of detecting a DDoS attack using the feature selected in a 5G network where a lot of IoT data is generated. Preprocessing through feature selection is useful and effective in detecting IoT DDoS attacks with low latency in 5G networks.

Thus, 5G should provide services to users with low latency. This requires ML-based automated intrusion detection systems to detect DDoS attacks at high speeds. Through experiments, this study confirmed that the feature selection process could have a significant effect on reducing the time complexity. In addition, compared with the large reduction in time required, the performance was maintained and improved. This shows that research related to feature engineering to detect large-capacity DDoS attacks in real time in 5G mobile networks is significant.

## 7. Conclusions

This study detected DDoS attacks using ML-based multiclass classification in a 5G mobile network environment. Subsequently, the effect of the feature selection method on the detection performance and time complexity was studied. For this purpose, an experimental 5G environment was constructed, hypotheses were made, and the 5G dataset was collected. In the experiment, the classification results using the features selected by the filter and wrapper methods were compared with those of a base model using all extracted features.

In the base model, which used all 55 features, the stacking algorithm showed the highest accuracy at 97.264%. When feature selection was applied, the number of features decreased by at least 25.45%, and the multiclass classification performance exceeded 82%. In particular, DT and RF increased the accuracy of the SFS (backward) method by 9.1% and 6.34%. In contrast, KNN increased the accuracy by 17.668% in the SFS (forward) method. The accuracy of the stacking algorithm was 97.183%, which was 0.083% lower than the accuracy when all features were used. In other words, compared to the large reduction rate of the number of features, the performance was maintained and improved. In addition, an experiment was conducted to confirm the change in detection and training time according to the increase in the size of the dataset. As the size of the dataset increased, training and detection times increased dramatically when all features were used. However, when feature selection was used, the training and detection time required for algorithms was maintained in seconds. As a result, as the size of the dataset increased, the difference in time complexity between the model using feature selection and the model using all features increased exponentially. Experiments showed that feature studies using feature selection are significant for improving DDoS detection performance and reducing time complexity. As a result, we confirmed the possibility of real-time DDoS attack detection via a feature selection process. Therefore, research related to feature engineering is required to detect large-capacity DDoS attacks with low latency in 5G mobile network environments.

Although this study established a 5G environment, only one gNB was built for the experiment. Therefore, in the near future, we will build two or more gNBs and establish an experimental environment by allocating gNBs by IP bandwidth. Subsequently, we plan to conduct feature-engineering-related experiments to detect DDoS attacks through multiple gNBs in real time. Simultaneously, we will conduct a feature study to detect more diverse types of DDoS attacks with low latency. In addition, 5G traffic was collected from the N3 network interface and entered from the gNB to the core in this study. However, because various network interfaces are used at the 5G core, a DDoS attack detection study that considers packets in other network interfaces is required.

The feature selection study for real-time detection of 5G mobile network DDoS attacks presented in this paper can be utilized in various applications. In particular, it can be utilized in a lightweight ML-based security function that can be operated in a 5G or 6G environment. Therefore, we plan to conduct feature engineering research that can be utilized in DDoS detection to achieve lower computational and time complexities. In addition, we plan to analyze the structure and procedure of the 5G core function and research a plan for the network function model that performs AI-based security detection in the 5G network standard structure.

## Figures and Tables

**Table 1:** The number of attack packets by attack type of the Kitsune dataset.

Attack Type	Attack Name	Benign Packets	Malicious Packets	Total Packets
Reconnaissance	OS Scan	1,632,151	65,700	1,697,851
	Fuzzing	1,811,356	432,783	2,244,139
Man in the Middle	Video Injection	102,499	2,369,902	2,472,401
	ARP MitM	1,358,995	1,145,272	2,504,267
	Active Wiretap	1,355,473	923,216	2,278,689
Denial of Service	SSDP Flood	2,637,662	1,439,604	4,077,266
	SYN DoS	7038	2,764,238	2,771,276
	SSL Renegotiation	2,114,919	92,652	2,207,571
Botnet Malware	Mirai	121,620	642,516	764,136

**Table 2:** Source IP and uplink TEID assigned per UE.

UE Numbers	Source IP	TEID	UE Numbers	Source IP	TEID
UE 1-1	192.168.2.1	2	UE 1-11	192.168.3.11	42
UE 1-2	192.168.2.3	6	UE 1-12	192.168.3.12	46
UE 1-3	192.168.2.7	10	UE 1-13	192.168.3.13	50
UE 1-4	192.168.2.13	14	UE 1-14	192.168.3.14	54
UE 1-5	192.168.2.15	18	UE 1-15	192.168.3.20	58
UE 1-6	192.168.0.110	22	UE 1-16	192.168.3.22	62
UE 1-7	192.168.100.5	26	UE 1-17	192.168.3.107	66
UE 1-8	192.168.100.222	30	UE 1-18	169.254.176.87	70
UE 1-9	192.168.3.1	34	UE 1-19	169.254.174.17	114
UE 1-10	192.168.3.7	38	UE 1-20	0.0.0.0	78

**Table 3:** Features extracted from each packet.

Protocol	List of Features				
ICMP (4)	type	code	checksum	checksum_status	
IGMP (5)	type	max_resp	checksum	checksum_status	num_grp_recs
TCP (17)	port	seq	seq_raw	urgent_pointer	
	ack	ack_raw	ack_nonzero	window_size	
	flags	flags_res	flags_urg	flags_ack	flags_push
	flags_syn	flags_fin	checksum	checksum_status	
UDP (5)	src_port	dst_port	checksum	checksum_status	length
IPv4 (10)	version	len	id	flags	frag_offset
	ttl	proto	checksum	src_ip	dst_ip
GTP-U (14)	flags	flags_e	flags_reserved	flags_payload	flags_version
	flags_s	flags_pn	ext_hdr_next	ext_hdr_length	
	message	teid	ext_hdr_pdu_ses_con_pdu_type		
	ext_hdr_pdu_ses_con_qos_flow_id				

**Table 4:** Multiclass classification performance with all 55 extracted features.

Algorithm	Accuracy	Precision	F1-Score	Recall	AUC
DT	81.289%	87.272%	87.258%	93.081%	94.868%
RF	86.537%	91.012%	90.709%	94.513%	95.911%
KNN	70.038%	78.863%	77.96%	84.879%	90.055%
Stacking	97.264%	97.609%	96.992%	96.47%	97.951%

**Table 5:** Multiclass classification performance using features selected through feature importance.

Algorithm	No. of Features	Accuracy	Precision	F1-Score	Recall	AUC
DT	5	80.037%	86.512%	85.654%	91.075%	93.733%
	10	83.611%	89.503%	89.048%	93.663%	95.294%
	55 (all)	81.289%	87.272%	87.258%	93.081%	94.868%
RF	5	86.255%	90.762%	90.572%	94.513%	95.897%
	10	91.069%	94.001%	93.605%	95.684%	96.784%
	55 (all)	86.537%	91.012%	90.709%	94.513%	95.911%

**Table 6:** Multiclass classification performance using the subset in 5 or 10 features selected through RFE.

Algorithm	No. of Features	Accuracy	Precision	F1-Score	Recall	AUC
DT	5	82.775%	88.696%	88.271%	92.427%	94.586%
	10	81.308%	87.188%	87.336%	93.113%	94.884%
	55 (all)	81.289%	87.272%	87.258%	93.081%	94.866%
RF	5	83.313%	89.442%	88.998%	93.771%	95.334%
	10	85.262%	90.029%	89.967%	94.247%	95.701%
	55 (all)	86.537%	91.012%	90.709%	94.513%	95.911%

**Table 7:** Multiclass classification performance using the feature subset selected through RFECV.

Algorithm	No. of Features	Accuracy	Precision	F1-Score	Recall	AUC
DT	41	82.976%	89.013%	88.524%	93.522%	95.188%
	55 (all)	81.289%	87.272%	87.258%	93.082%	94.868%
RF	39	86.705%	90.673%	90.629%	94.585%	95.967%
	55 (all)	86.537%	91.012%	90.709%	94.513%	95.911%

**Table 8:** Multiclass classification performance using the subset in 5 or 10 features selected in the forward or backward direction through SFS.

Algorithm	Direction	No. of Features	Accuracy	Precision	F1-Score	Recall	AUC
DT	Forward	5	88.455%	92.09%	91.925%	94.859%	96.201%
		10	86.733%	91.025%	90.818%	94.428%	95.875%
	Backward	5	86.733%	91.025%	90.818%	94.428%	95.875%
		10	88.687%	92.108%	92.132%	94.917%	96.244%
	-	55 (all)	81.289%	87.272%	87.258%	93.080%	94.868%
RF	Forward	5	90.12%	94.38%	93.627%	95.465%	96.595%
		10	90.077%	93.91%	93.185%	95.447%	96.585%
	Backward	5	92.026%	95.154%	94.457%	95.926%	96.95%
		10	89.86%	94.714%	93.606%	95.377%	96.527%
	-	55 (all)	86.537%	91.012%	90.709%	94.513%	95.911%
KNN	Forward	5	82.313%	85.874%	86.924%	92.773%	94.818%
		10	82.413%	85.932%	86.993%	92.823%	94.851%
	Backward	5	82.456%	85.539%	86.844%	92.772%	94.841%
		10	82.466%	85.524%	86.805%	92.607%	94.758%
	-	55 (all)	70.038%	78.863%	77.96%	84.879%	90.055%

**Table 9:** List of features used by the stacking algorithm.

No. of Features Used	Protocol	List of Features			
5	TCP (2)	seq_raw	window_size		
	IPv4 (2)	id	checksum		
	GTP (1)	teid			
10	TCP (4)	seq_raw	window_size	flags_syn	tcp
	UDP (1)	checksum			
	IPv4 (3)	id	checksum	len	
	GTP (2)	teid	length		

**Table 10:** Multiclass classification performance using 5 or 10 features selected through the frequency of the features selected for each classification algorithm used as the regression model of the stacking algorithm.

Features	Accuracy	Precision	F1-Score	Recall	AUC
5	97.106%	97.208%	96.997%	96.799%	98.113%
10	97.183%	97.442%	97.065%	96.724%	98.075%
55 (all)	97.264%	97.609%	96.992%	96.47%	97.951%

**Table 11:** Comparison of multiclass classification performances for the base model and the best model applying each feature selection method.

Algorithm	Method	Features	Accuracy	Precision	F1-Score	Recall	AUC
DT	-	55	81.289%	87.272%	87.258%	93.080%	94.868%
	Feature importance	10	83.611%	89.503%	89.048%	93.663%	95.294%
	RFE	5	82.775%	88.696%	88.271%	92.427%	94.586%
	RFECV	41	82.976%	89.013%	88.524%	93.522%	95.188%
	SFS (forward)	5	88.455%	92.09%	91.925%	94.859%	96.201%
	SFS (backward)	10	88.687%	92.108%	92.132%	94.917%	96.244%
RF	-	55	86.537%	91.012%	90.709%	94.513%	95.911%
	Feature importance	10	91.069%	94.001%	93.605%	95.684%	96.784%
	RFE	10	85.262%	90.029%	89.967%	94.247%	95.7%
	RFECV	39	86.705%	90.673%	90.629%	94.585%	95.967%
	SFS (forward)	5	90.12%	94.38%	93.627%	95.465%	96.595%
	SFS (backward)	5	92.026%	95.154%	94.457%	95.926%	96.95%
KNN	-	55	70.038%	78.863%	77.96%	84.879%	90.055%
	SFS (forward)	10	82.413%	85.932%	86.993%	92.823%	94.851%
	SFS (backward)	10	82.466%	85.524%	86.805%	92.607%	94.758%
Stacking	-	55	97.264%	97.609%	96.992%	96.47%	97.951%
	feature selection	10	97.183%	97.442%	97.065%	96.724%	98.075%

**Table 12:** Comparison of the results with similar related works.

Dataset Used	ML Algorithm	Feature Selection Method	Accuracy	F1-Score	Ref.
KDD Cup 1999, NSL-KDD (2009)	k-means++ and AdaBoost	Importance measurement using OOB of RF	92.62%	91.02%	[22]
Their own dataset	SVM	Filter	92.46%	90.43%	[23]
		Wrapper	92.15%	90.21%	
		Embedded	92.46%	90.6%	
	KNN	Filter	97.15%	96.92%	
		Wrapper	98.3%	97.7%	
		Embedded	96.3%	97.8%	
	ANN	Filter	92.28%	90.2%	
		Wrapper	91.44%	87.89%	
		Embedded	92.09%	89.06%	
	NB	Filter	95.7%	93.6%	
		Wrapper	94.87%	92.01%	
		Embedded	95.09%	93.18%	
5G dataset (Collected using MedBIoT)	KNN	Without feature selection	44.72%	26.57%	[20]
	SVM		50%	16.67%	
	RF		62.48%	58.86%	
	Stacking		62.65%	29.83%	

**Table 13:** Comparison of time complexity change according to dataset size change for the base model and the best model applying each feature selection method.

Algorithm	Selection Method	No. of Features	The Required Time (s)				
			91,000	182,000	273,000	364,000	455,000
DT	-	55	2.298	4.535	6.968	9.673	11.973
	Feature importance	10	1.177	2.479	3.627	4.661	5.823
	RFE	5	0.988	1.814	2.699	3.475	4.324
	RFECV	41	1.858	3.934	5.962	8.076	10.090
	SFS (forward)	5	0.705	1.298	1.931	2.501	3.147
	SFS (backward)	10	0.752	1.394	2.167	2.792	3.643
RF	-	55	7.106	13.865	21.069	29.293	37.363
	Feature importance	10	6.067	10.636	15.536	21.037	26.800
	RFE	10	5.757	10.152	14.969	20.133	25.806
	RFECV	39	6.669	12.926	19.823	27.007	34.637
	SFS (forward)	5	5.418	8.808	12.177	16.422	21.010
	SFS (backward)	5	5.357	8.774	12.450	16.352	20.611
KNN	-	55	141.467	585.745	1445.308	2889.444	4626.100
	SFS (forward)	10	6.659	13.214	22.522	29.400	39.097
	SFS (backward)	10	6.467	13.3	21.428	28.367	37.891
Stacking	-	55	316.492	1205.926	2807.596	5255.847	9243.293
	feature selection	10	25.529	45.827	68.820	91.241	109.952

## Data Availability

Not applicable.
